# Supplementary material for: Arl2 GTPase associates with the centrosomal protein Cdk5rap2 to regulate cortical development via microtubule organization
Source: PLoS Biol. 2024 Aug 13;22(8):e3002751. doi: 10.1371/journal.pbio.3002751 (PMC11321591; doi:10.1371/journal.pbio.3002751)

Raw Data → Western Blot and Co-IP:

Supplementary Figure 1A

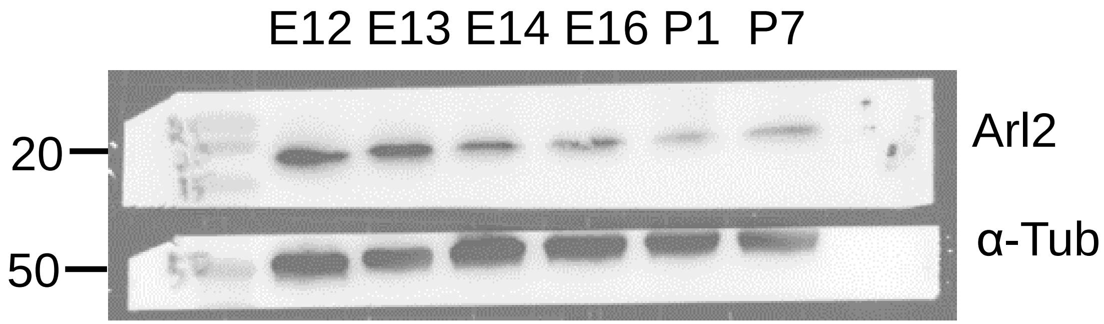

Supplementary Figure 1D

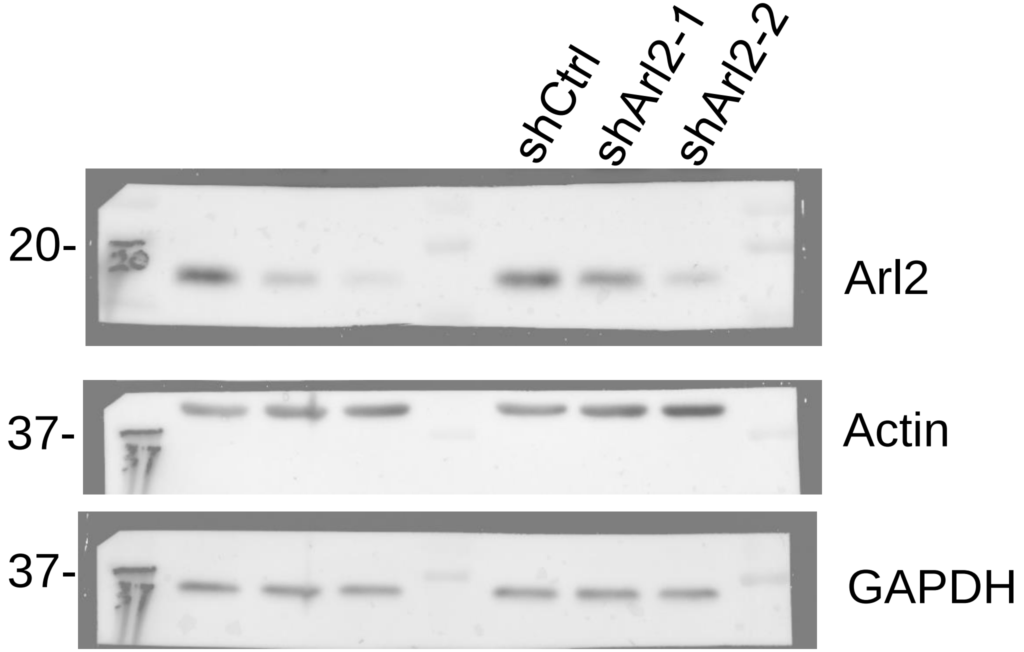

Figure 7F

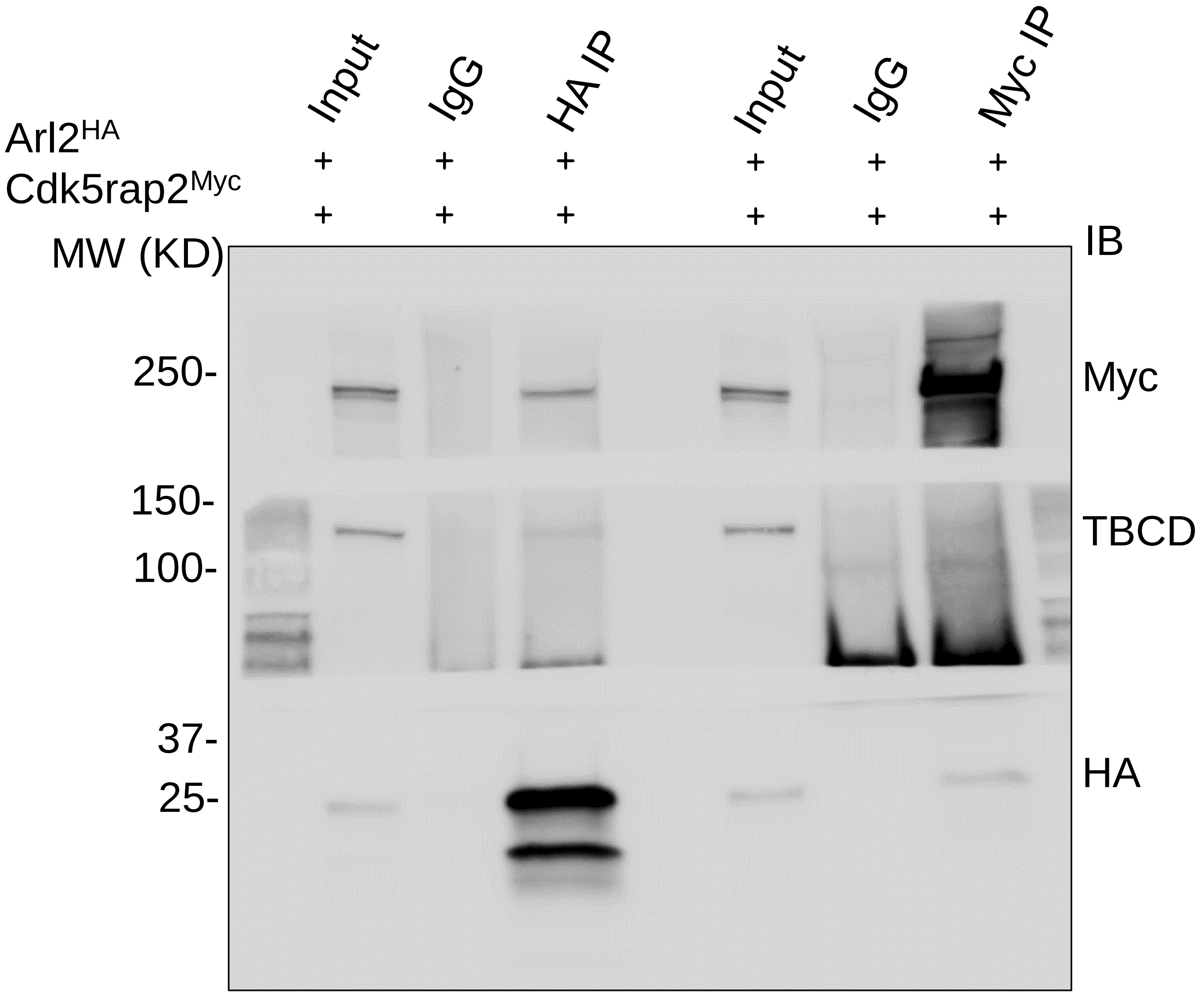
Input
IgG
HA IP
Input
IgG
Myc IP
Arl2HA
Cdk5rap2Myc
+ + + + + +
+ + + + + +
MW (KD)
IB
250-
150-
100-
37-
25-
Myc
TBCD
HA

Figure 8C:

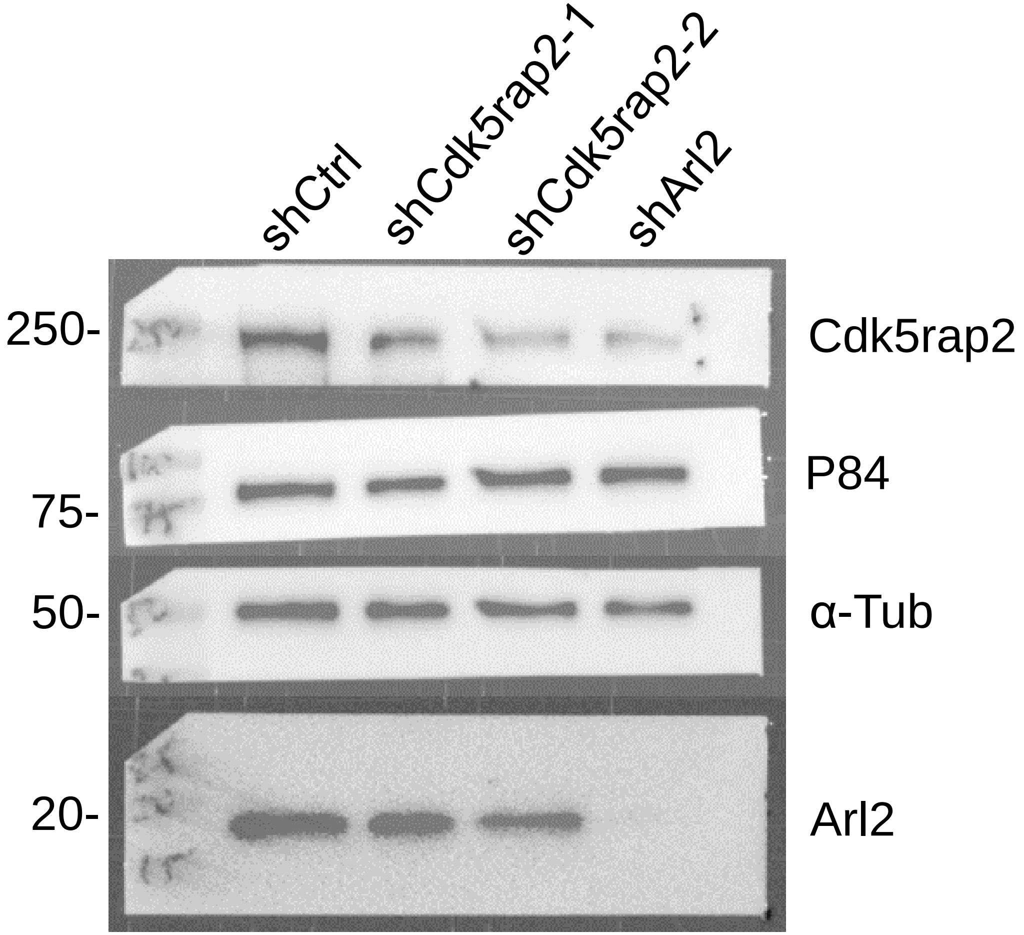

Supp Fig 7A

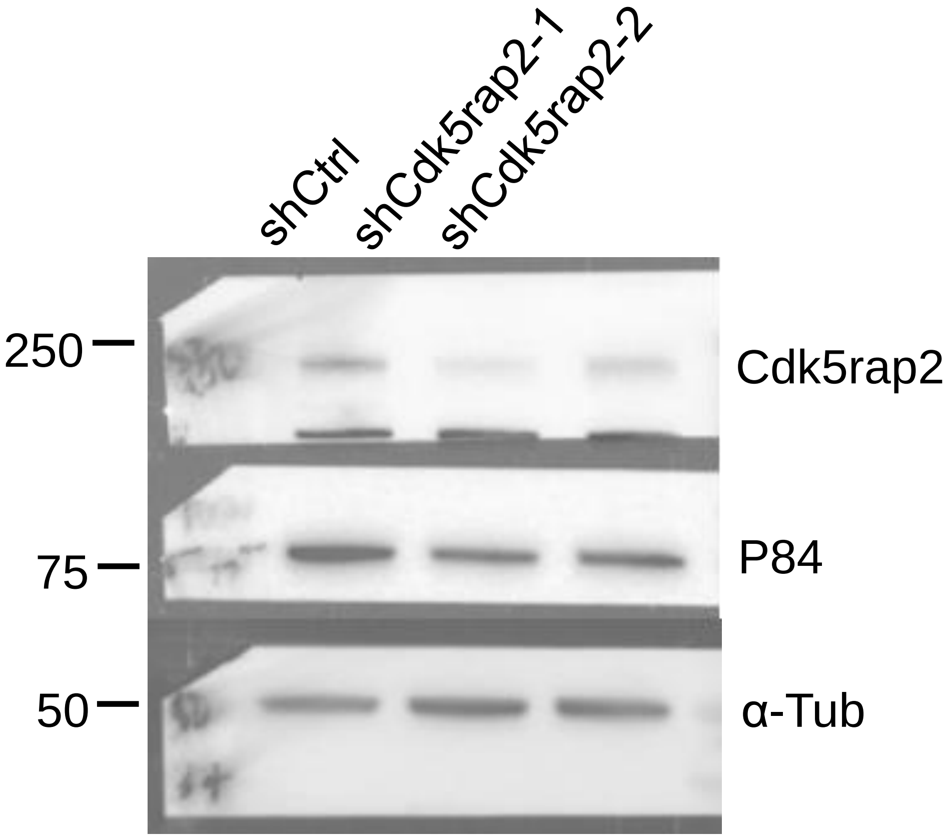

Supplement: S1 Raw Images — (PDF) [file pbio.3002751.s016.pdf]
