# Supplementary material for: Arl2 GTPase associates with the centrosomal protein Cdk5rap2 to regulate cortical development via microtubule organization
Source: PLoS Biol. 2024 Aug 13;22(8):e3002751. doi: 10.1371/journal.pbio.3002751 (PMC11321591; doi:10.1371/journal.pbio.3002751)
Supplement: Attachment — Submitted filename: Response letter_Arl2 24-05-2024 HY final.pdf [file pbio.3002751.s017.pdf]

Point-by-point response to reviewers' comments

Reviewer #1:

This manuscript by Dong-Lian Ma focuses on the role of Arl2 (ADP ribosylation factor-like GTPase 2) in mouse cortical neurogenesis and its molecular interactions. The authors, who have discovered Arl2 is involved neural progenitor cell divisions, found that this is also the case. They discovered that mouse Arl2 plays a new role in corticogenesis via regulating microtubule growth, but not mitochondria functions, and that it molecularly interacts with centrosomal protein, Cdk5Rrap2, whose loss is known to cause microcephaly. The authors revealed Arl2 functions upstream of Cdk5Rrap2. Thus, the authors propose that Arl2 plays critical role in mammalian corticogenesis via controlling centrosome and/or microtubule functions.
The manuscript includes fascinating findings regarding molecular and cell biological functions of Arl2 in mammalian brain development and microtubule dynamics that are required for cell migration and divisions of neural stem cell (NSC)s. The reviewer points out a couple of points that are important and necessary to be improved to be published in PLOS Biology.

We thank the reviewer's strong endorsement of our manuscript and constructive comments.

1. Line 119-127
Arl2 KD certainly causes retention of NSCs. The authors indicate that reduced proliferation of NCS in the VZ and SVZ as well as the inhibition of cell migration into the cortical plate. However, no clear test has been done to clarify whether defective migration of VZ/SVZ cells is caused by either "neuron"s migration or defective differentiation from progenitors to "neurons" leading to their retention in the VZ. So it seems to be overstated that Arl2 knock-down (KD) data suggest "neuronal" migration defect in this version of the manuscript.

We have now performed the following three experiments to clarify whether defective migration of VZ/SVZ cells is caused by neuronal migration and/or defective differentiation from progenitors to neurons, leading to their retention in the VZ *in vivo*.

1. We examined NeuroD2, a neuronal marker found in newly born neurons in IZ and immature/mature neurons in CP and quantified the proportion of NeuroD2+GFP+ cells in IZ 3 days after IUE. At E16, 3 days after IUE, we observed a significant decrease in the proportion of NeuroD2+GFP+ cells in the shArl2 group (42.06 ± 4.42%) as compared to the control group (75.49 ± 3.77%). This data suggests a notable impairment in the differentiation of NSCs due to Arl2 KD *in vivo*. We have added this new data to the revised manuscript as Figure2A-B on Page 8.

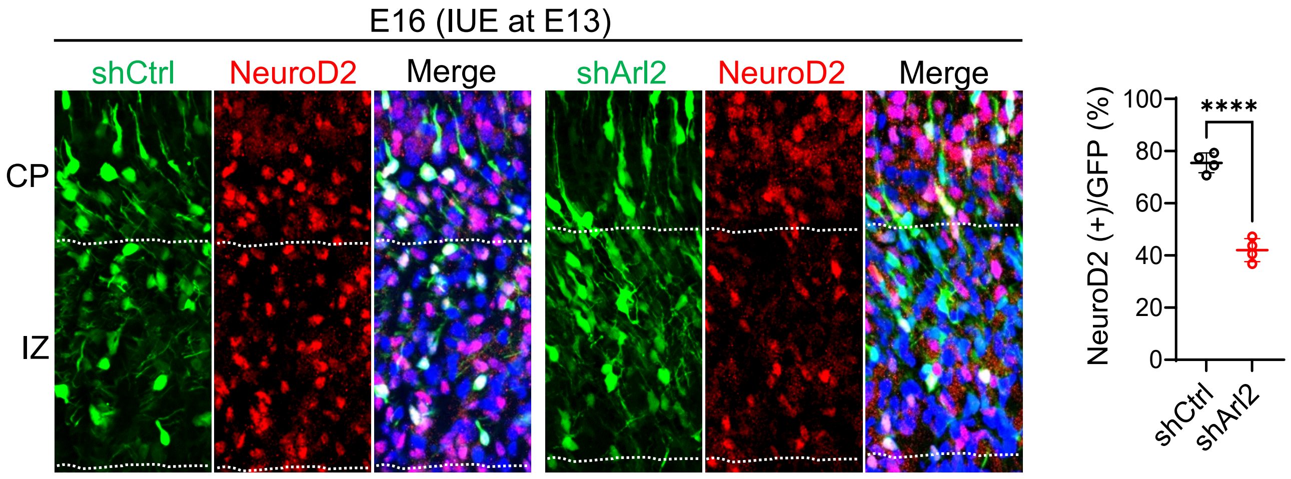

2. We have also quantified the migration distance of NeuroD2+GFP+ cells in the mouse brain 4 days after IUE. We find that knockdown Arl2 resulted in significantly less migration of NeuroD2+GFP+ cells (104.3 ± 13.05 μm) as compared to the control group (135.8 ± 9.81 μm), suggesting a defect in neuronal migration. We have added this new data to the revised manuscript as Figure 2G-H on page 8-9.

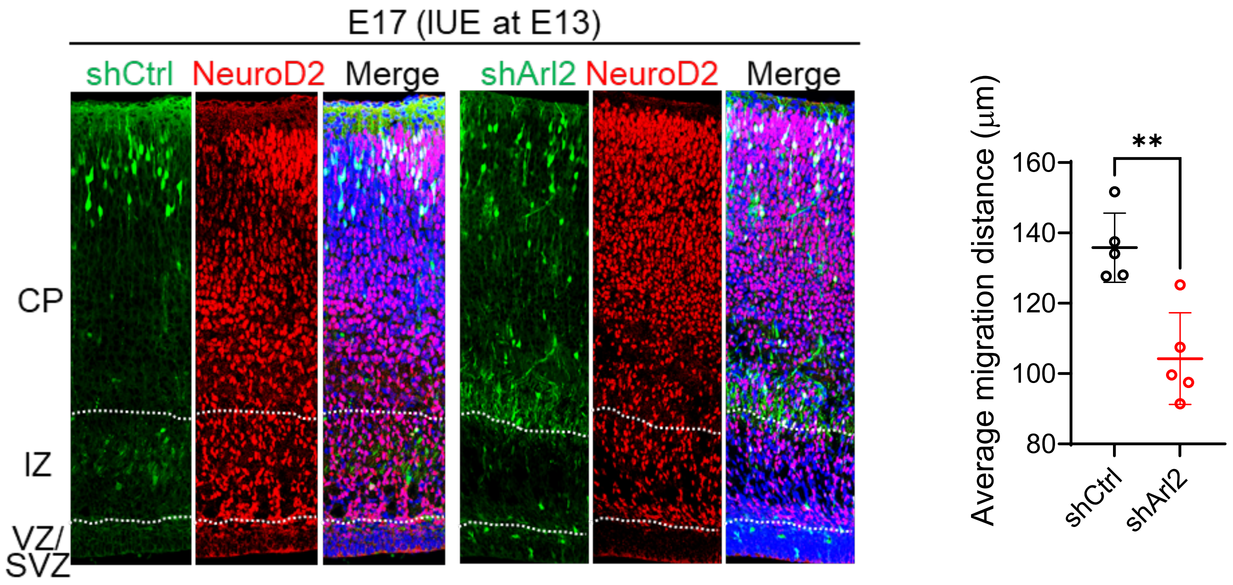

3. We performed Arl2 KD in mouse primary neurons *in vitro* and observed defects in neuronal morphology, as fewer and shorter neurites were seen in these neurons as compared to control (images below). We also observe that the total intersection number as measured by Sholl's analysis was significantly reduced in shArl2 (22.78 ± 5.04) as compared to control (64.17 ± 2.62). This neurite outgrowth defects in Arl2 KD neurons further supported their neuronal

migration defects. We have added this data as Supplementary Figure 2A-D on page 8-9 of the revised manuscript.

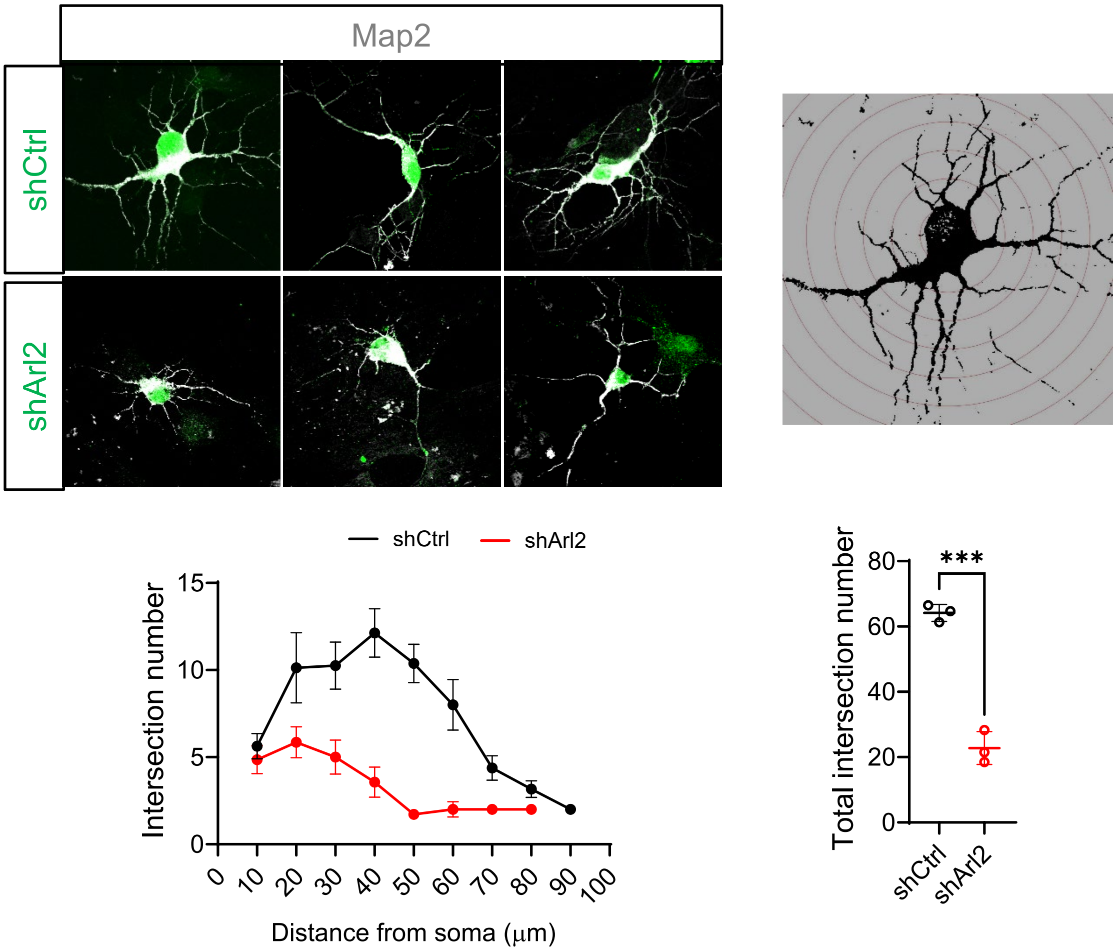

2. Line 167-168
The authors state that overexpression of both Arl2T30N and Arl2Q70L results in a similar phenotype in neuronal migration as Arl2 KD. However, the reviewer could not find experiments in this manuscript to examine whether Arl2 KD cause cell cycle arrest (increased PH3+) and/or cell death. The reviewer considers that this is a missing but important data.
3. Line 169-181
AS for two Arl2 mutants, Arl2[T10N] and arL3 Q70L, the authors clearly observe cell cycle arrest (an increase in PH3+ cells; SupFig.2D in vivo) and extensive cell death in the VZ (SupFig.2A, C, for in vitro, and SupFig.3B for In vivo) and state "These data suggest that the migration and proliferation defects (mitotic arrest) are observed in Arl2 mutants, which eventually leading to cell death".
The reviewer does not understand why the authors do not test the degree of cell death caused by Arl2KD. These data by using Arl2 KD will bring us much straightforward and consistent interpretation about the results by Arl2 perturbation.

As suggested by the reviewer, we have now extended our analysis on cell cycle arrest and cell death to Arl2 knockdown.

1. To understand the effects of Arl2 knockdown (KD) on the cell death in the mouse cortex during cortical development *in vivo*, we examined the cell death/apoptosis marker, active caspase-3. We find that at E14, 1 day after IUE, there was a significant increase in the proportion of Caspase-3+ GFP+ cells in the VZ and SVZ (15.7 ± 1.85%) as compared to control (2.65 ± 0.80%), suggesting an increase in cell death *in vivo*. We have added this data to the revised manuscript on page 15 and Supplementary Figure 2E-F.

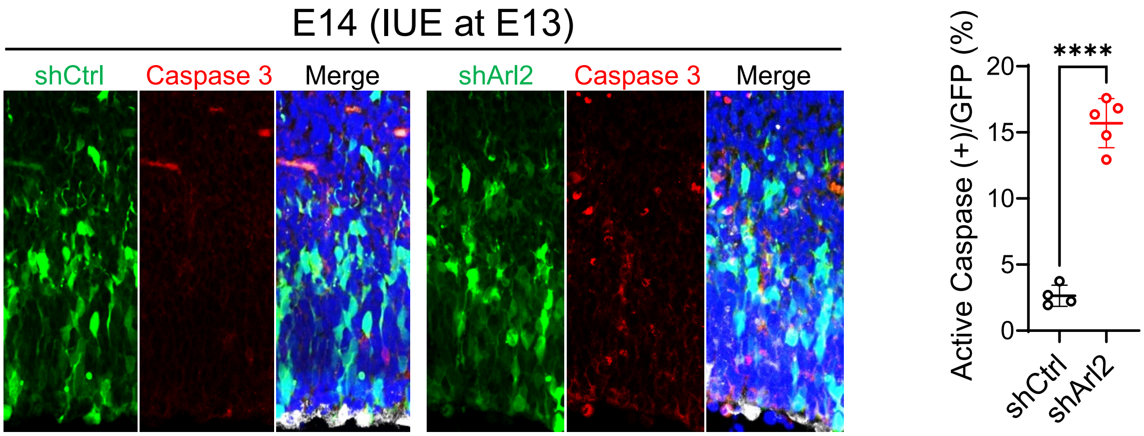

2. We have also examined whether Arl2 knockdown (KD) affects cell death in mouse neural progenitor cells (mNPCs) *in vitro*. We find that there was a significant increase in the proportion of caspase-3+ GFP+ cells in the Arl2 KD mNPCs (25.86 ± 3.26%) as compared to control (9.64 ± 0.86%), suggesting an increase in cell death *in vitro*. We have added this data as Supplementary Figure 1F, H on page 5 of the revised manuscript.

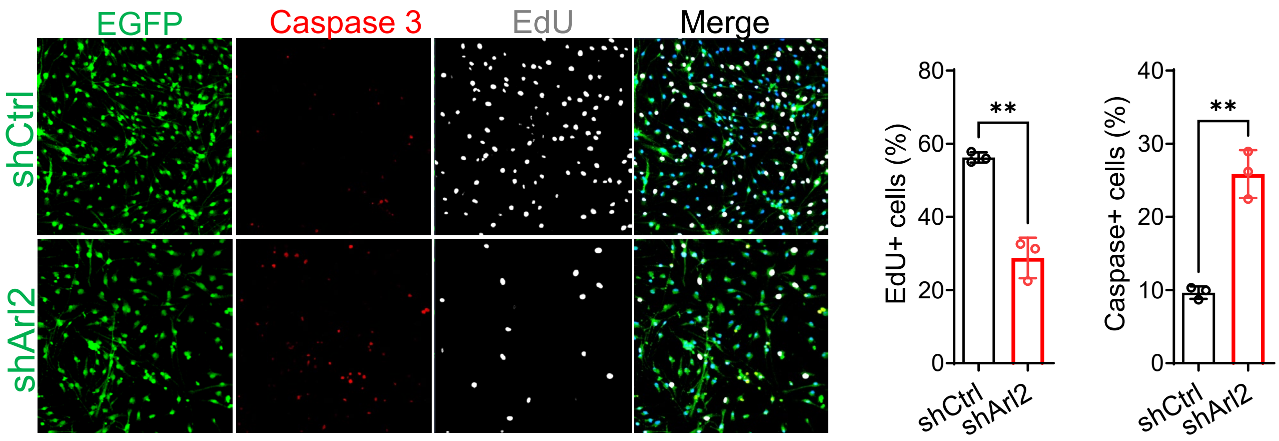

3. We have now performed live imaging of mNPCs *in vitro* using the Viafluor-647 live cell microtubule staining kit (Biotium, #70063) to examine whether Arl2 affects mNPC proliferation. We observe that KD Arl2 mNPCs caused a significant increase in the mitotic

duration as compared to the control (shCtrl = 1.32 ± 0.21 hours, n = 9 vs. shArl2 = 6.55 ± 1.52 hours, n = 7), suggesting an additional defect in the cell cycle progression caused by Arl2 KD.

Taken together, our live imaging, *in vivo* and *in vitro* Caspase-3 results suggests that the proliferation defects observed in Arl2 KD are possibly due to mitotic defects, eventually leading to cell death. We have now added this data to the revised manuscript on page 15, Figure 4A-B and Movie S3.

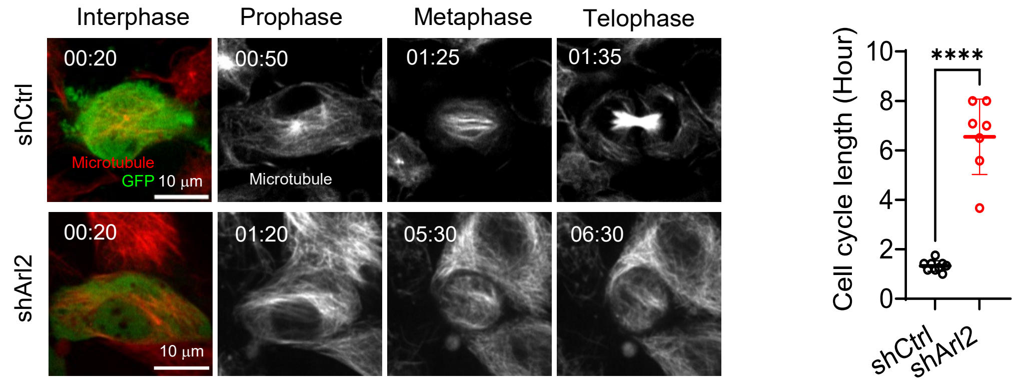

4. Line 182-194
A main conclusion of this study is that Arl2 dysfunction results in a defective migration of "neuronal" cells; the authors state that "the expression of NeuroD2, a neuronal marker found in immature neurons, is significantly increased in Arl2WT (30.77 ± 2.93%) but dramatically reduced in Arl2Q70L (6.75 ± 2.69%) 3 days after IUE as compared to control (20.57 ± 1.36%) (Supplementary Figure 3D-E). Taken together, Arl2 dysfunction resulted in a defective migration of neuronal cells."
As far as the reviewer understand correctly, in this study, the authors did not show evidence that defective migration occurs for "differentiated" cells, namely "neuronal cells". The reviewer considers that it would be better to exclude the possibility that the production of neurons is inefficient from progenitors or that immature neurons die before migration. My concern comes from the results regarding NeuroD2+ cells rather looking like that neuronal differentiation are diminished.

As we responded to question #1 above (page 1-3 of this response letter), we have provided additional data to show that upon Arl2 KD, both neuronal differentiation and neuronal migration are compromised.

5. Line 225-241
The authors claim that loss of Arl2 led to a shift of symmetric division to asymmetric division of mNPCs and alters the mNPC differentiation.
This claim is based on observing perturbed spindle orientations by Arl2 KD, and the authors state that Arl2-depleted NPCs divides asymmetrically frequently than symmetrically as compared to control; line 230 "At E14, one day after IUE, wild-type radial glial cells in the VZ can undergo both symmetric and asymmetric divisions, depending on the plane of division (Figure 4A-C)."

The reviewers did not agree this claim from two points of view.
First of all, at E14, the stage when layer 4 neurons are generated, most NSCs (RG) divide asymmetrically in their fate (self-renewal vs. differentiation).
Secondly, it has been established that division plane orientation of cortical NSCs is nothing to do with the division styles self-renewal or differentiation of NSCs (Konno et al. 2008, Shitamukai et al. 2011, Morin & Bellaïche 2011). Rather, cleavage plane orientation is related with the generation of migrating NSCs. If the cleavage plane bypasses the apical membrane, one daughter (without the apical attachment) becomes a migrating self-renewing NSC with another differentiating daughter (with the apical attachment); if the cleavage plane bisects the apical membrane bisects, one daughter becomes a radial glia and the other an differentiating intermediate daughter.
Given this, it is reasonable that the NSC population in the VZ will not drastically change under the Arl2 KD condition, where a majority of cells (including NSCs) are trapped in the VZ and SVZ.
If the authors want to keep this part in the manuscript, the reviewer also strongly recommends to examine whether the fate of cortical NSCs is precisely followed in the wild type to see whether it depends cleavage orientation as a control and then compare the result with the situation of Arl2 KD. Also, the cleavage plane is defined in the 3D tissue structure. Therefore, it is strongly recommended to take a protocol by which cleavage planes are measured considering the 3D space.

We have taken into consideration the reviewer's valuable comments as well as the editor's suggestion and have removed the section claiming that 'loss of Arl2 led to a shift of symmetric division to asymmetric division of mNPCs and alters the mNPC differentiation' from our manuscript. Our manuscript now focuses on the differentiation outcome and migration effects of Arl2. This change does not affect our main conclusion of this manuscript.

Reviewer #2:

In this study, Ma et al. describe a novel role for Arl2 in mouse cerebral cortex development. The authors, using knockdown and overexpression strategies either in vitro or in vivo, show that Arl2 is important for neural progenitor proliferation and neuronal migration during corticogenesis in mice. These effects are linked to its activity on microtubules via the centrosomal protein Cdk5rap2. The manuscript presents new information that will be of interest to developmental neurobiologists. The experiments are well conducted and convincing. However, several points need to be addressed before publication.

We thank the reviewer's strong endorsement of our manuscript and constructive comments.

Major points:

1. The expression pattern of Arl2 in the embryonic cerebral cortex must be described either at the RNA or protein levels on cortical sections. This will help to understand the functions of Arl2 in the developing cortex.

As suggested by the reviewer, we have now analyzed Arl2 protein levels by Western Blot in mouse cerebral cortical tissue isolated from E12, 13, 14, 16, P1, P7 during the mouse cerebral cortical development. We find that Arl2 protein levels are highest at embryonic stages (E12, 13, 14, 16) as compared to the postnatal stages (P1, P7). This is consistent with the role of Arl2 during the mouse cortical development. This data has now been added as Supplementary Figure 1A-B and Page 5 of the revised manuscript.

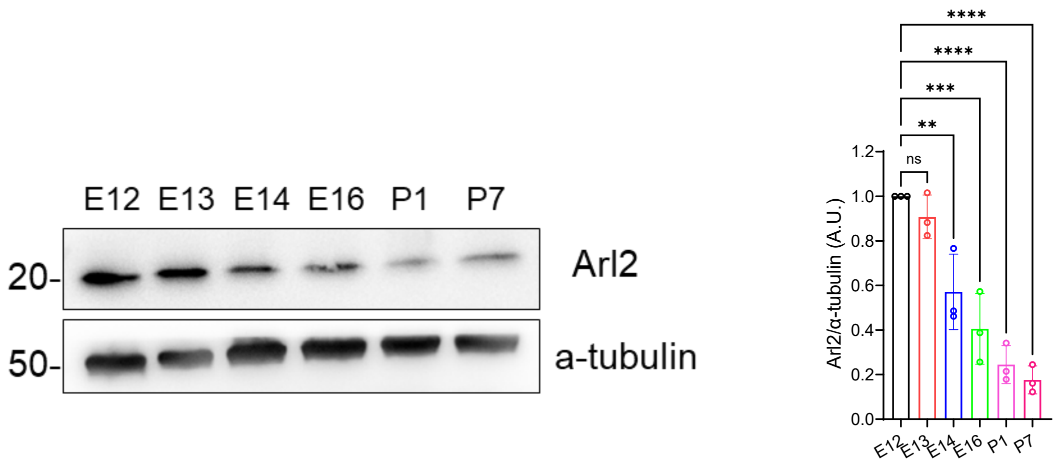

2. It is not clear whether the effects on migration are linked to the effect on cell cycle progression. Indeed, a delay in cell cycle progression could explain the reduction of cells reaching the cortical plate at a specific time point. The authors should determine whether Arl2 has a direct role on migration (for example by knocking down Arl2 only on postmitotic neurons or by performing live imaging to visualize migrating neurons). It would help also to look at the morphology of the migrating neurons directly in sections.

We have now performed two additional experiments to substantiate that Arl2 has a direct role in migration.

1. We examined NeuroD2, a neuronal marker found in newly born neurons in IZ and immature/mature neurons in CP and quantified the migration distance of NeuroD2+GFP+ cells in the mouse brain 4 days after IUE. We find that knockdown Arl2 resulted in significantly less migration of NeuroD2+ GFP+ cells (104.3 ± 13.05 μm) as compared to the control group (135.8 ± 9.81 μm), suggesting a defect in neuronal migration. We have added this new data to the revised manuscript as Figure 2G-H and page 8-9.

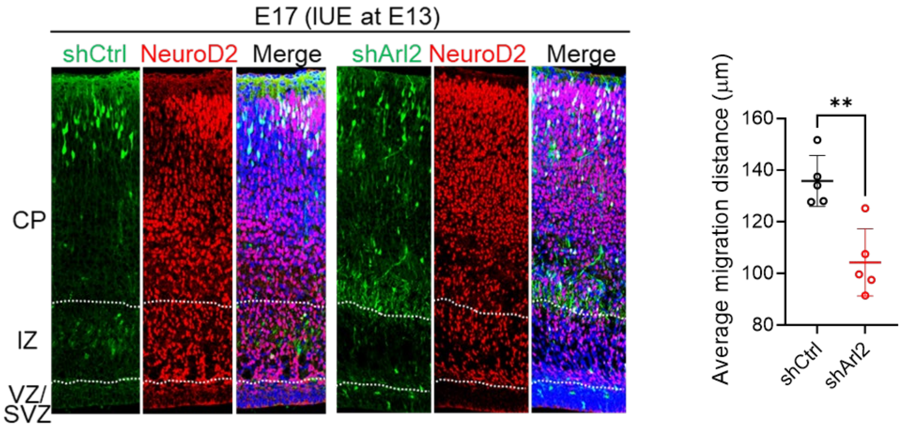

2. We have also examined the effect of Arl2 KD in mouse primary neurons *in vitro* as suggested by the reviewer. We find that Arl2 KD dramatically affected neuronal morphology, as fewer and shorter neurites were observed in these neurons as compared to control (images below). We also observe that the total intersection number as measured by Sholl's analysis was significantly reduced in shArl2 (22.78 ± 5.04) as compared to control (64.17 ± 2.62). This suggests that neuronal migration defects caused by Arl2 dysfunction is possibly due to its role in regulating neurite outgrowth. We have added this data as Supplementary Figure 2A-D on page 9 of the revised manuscript.

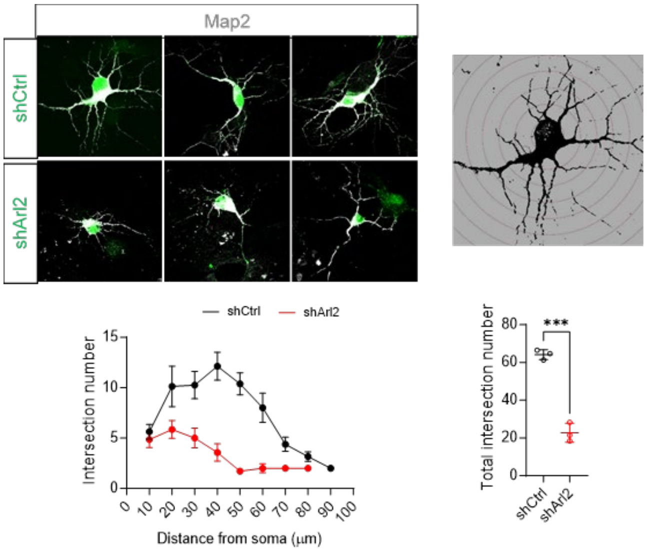

3. The number of n is sometimes low (n=3). It should be mentioned whether the three embryos used for quantification come from same or different litters.

We have now increased our sample size and N values to 4-5.

Each N value (one embryo) that is used for quantification comes from a different litter. We have updated this information in the Materials and Methods on page 37.

Minor points:

1. The reading is sometimes difficult because the figures are not cited in an ordered manner. For example the mutant Arl2K41R appears on Figure 2 but it is described in the text after figure 4. Similarly, some figure panels are not commented in the text such as Tbr2, Pax6 or Tbr1 stainings in Figure 1.

We thank the reviewer for pointing this out. We have now edited the text to match the figures and have made sure that all figure panels are called out in the text.

2. The two mutants, dominant negative and dominant active of Arl2, give similar phenotypes. This should be discussed.

We have now added the following discussion on why the two mutants, dominant negative and dominant active of Arl2, give similar phenotypes (page 32-33).
"Why overexpression of $Arl2^{Q70L}$ and $Arl2^{T30N}$ mutant forms exhibited the same phenotypes in these processes in mNPCs is unknown. In photoreceptor cells, overexpression of $Arl2^{Q70L}$ binds to ARL2BP and sequester its function, resulting in shortening of cilia in these cells, a phenotype similarly observed in Arl2 KD (Wright, Loskutov et al. 2018). It remains to be tested whether the phenotype induced by overexpression of $Arl2^{Q70L}$ in mNPCs is due to sequestering other proteins important for microtubule functions such as ARL2BP."

Reviewer #3:

This manuscript presents a mechanistic link between Arl2 and Cdk5rap2 and neuronal progenitor proliferation and neuronal migration in the developing mouse cortex. The authors have very carefully conducted a thorough set of experiments to support their hypothesis. Using a combination of in utero electroporations, shRNA knockdown, overexpression of Arl2 mutant constructs and primary neural cultures they report a novel finding that Arl2 is important for recruitment of both gamma tubulin and Cdk5rap2 to the centrosome and that the lack of this recruitment leads to increase in mitotic arrest in the Arl2 mutants which is rescued by Cdk5rap2 overexpression. In addition, they showed that Arl2 and Cdk5rap2 interact using multiple methodologies from in silico methods to proximity ligation assays and immunoprecipitation of overexpressed constructs. My comments see below are minor because I consider the authors have done outstanding work on supporting their claims and they provide important insights into the cell biology of neurogenesis.

We thank the reviewer for the overall enthusiasm on our manuscript and constructive comments.

1- In the abstract they only refer to the interaction between Arl2 and Cdk5rap2 as if it was only done in silico this should be revised to reflect that the authors used additional methods to prove the interaction between these two proteins.

We have now added 'Moreover, Arl2 physically associates with Cdk5rap2 by *in silico* prediction using AlphaFold Multimer **which was validated by co-immunoprecipitation and proximity ligation assay**'' to reflect that we have used additional methods to prove the interaction between Arl2 and Cdk5rap2 as suggested by the reviewer.

2- Across all figures where there are microscope images I recommend that they put a scale bar in each figure not just in one of the figures in the panel

We have now added a scale bar to each of the figures in the panel.

3- For all figures in which the cortex is being shown I suggest they show an enlarged image for when they want to show colocalization

We have now shown a zoomed in image for all images depicting colocalization within the cortex.

4- They have an interesting finding that when Arl2 is knockdown the total levels of Cdk5rap2 are also knockdown they attribute this to the lack of CDk5rap2 localization to the centrosome which leads to a loss of stability? I think it would be important for them to discuss this a little bit more on the discussion because their western blots are from total cell extract. Do they know if they use a protease inhibitor can they restore the levels of Cdk5rap2 even if its localization to the centrosome is still lost? I think that being able to separate these two cellular effects would be

important, is it that Arl2 somehow regulates the levels of Cdk5rap2 and that is why there is none in the centrosome or are those two separate from each other. Alternatively a good discussion on this topic would be important to include.

We have treated the mNPCs with a proteosome inhibitor (MG132) with DMSO as control. However, Cdk5rap2 levels were not restored by MG132 treatment in western blot and immunostaining (Figure below). Currently, how Cdk5rap2 protein turnover is regulated, for example via proteasome- or lysosome-mediated degradation, is unknown. Further study is required to investigate how Arl2 regulates Cdk5rap2 protein levels. We have included a discussion on this point in the revised manuscript (page 33).

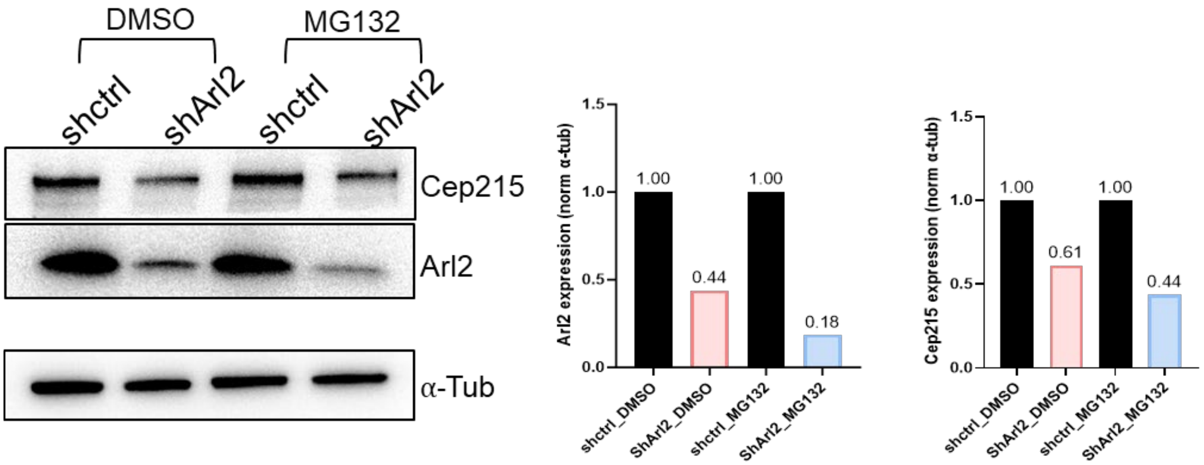

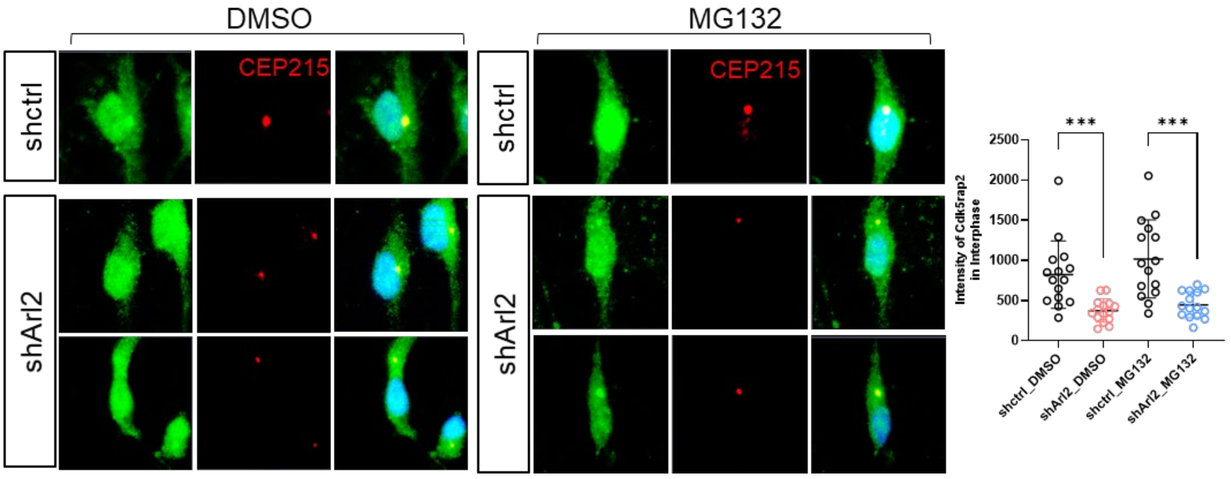

5- For the EB1 studies the P values are out of the total number of measurements or from the comparison of the mean values? There is a lot of overlap between the different groups. In addition, can the authors discuss if their changes in EB1 could be showing changes in microtubule dynamics since this is a marker of growing tips of microtubules

For our EB3 studies, the P values are from the total number of measurements and not from the comparison of mean values.

The overlap between the groups is possible since each point on the graph depicts a single comet. Since there is a variation in velocity, orientation and number of the EB3 comets within the control group itself, it is possible that we would find some comets within the mutant groups which may have similar velocity or number of comets as that of control.

Microtubules have a fast growing plus end and a slow growing minus end, which contribute to its dynamics. EB3-GFP has been widely used to track microtubule dynamics, as it marks the microtubule growth and dynamics by binding to the plus-ends of microtubules. EB3-GFP velocity and density monitored in live imaging are good representations for microtubule dynamics (Wimmer and Baffet 2023). We have included a short description about the rational using EB3-GFP in the revised manuscript (page 17).

References for the response letter

Wimmer, R. and A. D. Baffet (2023). "The microtubule cytoskeleton of radial glial progenitor cells." Curr Opin Neurobiol **80**: 102709.

Wright, Z. C., Y. Loskutov, D. Murphy, P. Stoilov, E. Pugacheva, A. F. X. Goldberg and V. Ramamurthy (2018). "ADP-Ribosylation Factor-Like 2 (ARL2) regulates cilia stability and development of outer segments in rod photoreceptor neurons." Sci Rep **8**(1): 16967.
