# Supplementary material for: Arl2 GTPase associates with the centrosomal protein Cdk5rap2 to regulate cortical development via microtubule organization
Source: PLoS Biol. 2024 Aug 13;22(8):e3002751. doi: 10.1371/journal.pbio.3002751 (PMC11321591; doi:10.1371/journal.pbio.3002751)

Response to Reviewers

Specifically, in response to Reviewer 2's comment 1, you show a western blot, but the reviewer specifically asked to see the expression in cortical sections, as its distribution within the tissue is the main interest here. In case the antibody is not good enough for IHC, please at the very least show a re-analysis of some existing scRNA-seq data to show which cell types Arl2 is most expressed in.

<u>Our response:</u>

Unfortunately, anti-Arl2 antibodies did not work for IHC, precluding us from analyzing Arl2's distribution in cortical sections. As suggested by the Editor, we re-analyzed a published scRNA-seq dataset, in which the embryonic mouse brain each day between E7 and E18 was profiled (Gioele La Manno, et al., Nature 2021). Our analysis (figure below) suggests that among various cell types in the developing mouse brain, Arl2 has the highest expression in radial glial cells, similar to Sox2, a neural stem cell marker. Arl2 also has high expression in neurons but a low expression level in glial cells and other cell types.

Taken together, Arl2 is most expressed in radial glial cells (neural stem cells) and neurons in the mouse developing brain, which is consistent with the role of Arl2 in the proliferation of neural stem cells and neuronal migration during cortical development. This new result has been included in Supplementary Figure 1C, page 5 of the revised manuscript.

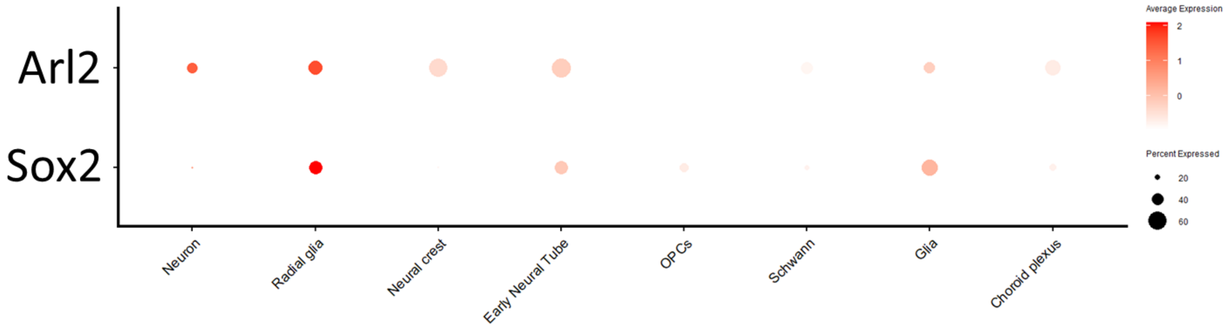

Supplement: Attachment — Submitted filename: Response to Reviewers 14 June 2024.pdf [file pbio.3002751.s018.pdf]
